# Ameliorative effects of crocin on the inflammation and oxidative stress-induced kidney damages by experimental periodontitis in rat

**DOI:** 10.22038/ijbms.2021.55875.12499

**Published:** 2021-06

**Authors:** Zeynep Erdemli, Mehmet Erman Erdemli, Mehmet Gul, Eyup Altinoz, Semir Gul, Gulhan Kocaman, Elif Kayhan Kustepe, Harika Gozukara Bag

**Affiliations:** 1 Inonu University, Faculty of Medicine, Medical Biochemistry Department, Malatya, Turkey; 2 Inonu University, Faculty of Medicine, Histology and Embryology Department, Malatya, Turkey; 3 Karabuk University, Faculty of Medicine, Medical Biochemistry Department, Karabuk, Turkey; 4 Karabuk University, Faculty of Dentistry, Periodontology Department, Karabuk, Turkey; 5 Inonu University, Faculty of Medicine, Biostatistics Department, Malatya, Turkey

**Keywords:** Crocin, Inflammation, Kidney damage, Oxidative stress, Periodontitis, Rat

## Abstract

**Objective(s):**

The present study aimed to investigate the effects of periodontitis on kidneys and the protective role of crocin in periodontitis-induced kidney damage.

**Materials and Methods:**

Ethics committee approval was obtained and 30 Wistar rats were randomly divided into 3 groups of 10 rats: Control (C), Periodontitis (P), and Periodontitis + Crocin (P + Cr). After the treatments, rat kidney tissues were incised under anesthesia and blood samples were collected. Biochemical and histopathological analyses were conducted on the samples.

**Results:**

Malondialdehyde (MDA), total oxidant status (TOS), and oxidative stress index (OSI) increased in P group rat kidney tissues; urea, creatinine, Tumor necrosis factor-α (TNF-α), interleukin-6 (IL-6), and interleukin 1β (IL-1β) levels increased in the serum; glutathione (GSH), superoxide dismutase (SOD), catalase (CAT) and total antioxidant status (TAS) levels were reduced in rat kidney tissues, and renal histopathology deteriorated. In the P + Cr group, we observed improvements in biochemical and histopathological parameters when compared with the P group.

**Conclusion:**

Periodontitis (P) led to deterioration in oxidative stress parameters and histopathology by increasing the oxidants in kidney tissue. P also led to inflammation in the blood of the rats. Periodontitis + Crocin (P + Cr) administration alleviated the effects of P due to powerful antioxidant anti-inflammatory properties. Cr could be employed as a protective agent in P-induced inflammation and oxidative damage.

## Introduction

Periodontitis (P) is initially observed on tooth surfaces and the dental plaque between teeth. It includes a group of bacteria where dental plaque metabolites trigger inflammation, leading to tissue loss in gums (1, 2). In fact, when there is no dental plaque, there is no periodontitis. Plaque is soft at first, over time, it hardens and forms tartar, leading to a dangerous chain reaction. Tartar roughens the tooth surface and creates an ideal environment for bacterial growth (3, 4). The immune system responds to the development with gingivitis, a superficial inflammation. This response prevents bacteria from penetrating deeper tissues for a period. But after a while, this natural barrier cannot stand against the constant bacterial attack. When no action is taken, bacteria enter the deeper tissues and inflammation turns chronic (5). In chronic inflammation, the acute inflammatory response leads to accumulation of pro-inflammatory mediators (Interleukin-1 (IL-1), Interleukin-6 (IL-6), tumor necrosis factor-alpha (TNF-α)) in tissues and tissue damages since the process was not inhibited or delayed. The inflammation could prevail despite tissue damage. It eventually leads to immune system dysregulation, where the inflammation reaction damages the tissues, leading to P. P bacteria and inflammatory agents could penetrate the bloodstream through the gums and cause further damage. Thus, untreated P affects not only oral health but sometimes the whole body (6-8). Various chronic and acute inflammation may trigger an inflammatory response in the kidney (9). P may lead to a decline in kidney functions (10) since P contributes to overall systemic inflammatory load, (8, 11) which in turn may lead to a decline in kidney functions (12), and P bacteria and associated products could penetrate the bloodstream, (13) possibly leading to the kidney epithelial damages (14, 15). Prolonged low-level inflammation could lead to chronic kidney diseases. Especially in final-stage renal failure patients under dialysis, the systemic circulation pro-inflammatory and anti-inflammatory cytokine levels were reported to increase significantly due to reduced renal clearance and based on the repair of degenerated cells (16-18).

Saffron basically consists of 4 active compounds. These compounds are crocin (Cr), crocetin, picrocrocin, and safranal (19-21). Cr, the active component in saffron shows anti-inflammatory, anti-carcinogenic, antidepressant, anti-neuropathic, anti-oxidant properties, and is utilized both in folk and modern medicines (22-27).

The present study aimed to investigate the protective effects of Cr on the kidney tissues of P model rats. This was the first study on the topic. There were only a few studies on the effects of P on kidneys in the literature. The effects of P on the kidney and the protective role of Cr are studied for the first time in the current research.

## Materials and Methods

***Experimental animals***

The study was conducted with 30 adult female Wistar rats that weighed 225−250 g. The rats were procured from Inonu University, Faculty of Medicine, Experimental Animals Production and Research Center (INUTF-DEHUM). The ethics committee approval was obtained from the Inonu University experimental animal ethics committee (2016/A-107). During the study, the rats were kept in cages where the temperature was maintained at 21 °C and humidity was 55−60% under 12 hr light (08:00 −20:00), 12 hr dark cycle. A soft diet was fed to the rats in the study. Fresh drinking water was supplied, and cages were cleaned daily. Animals were allowed to eat and drink water *ad libitum*.

***Experimental design***

Experimental periodontitis was induced in the experimental groups around the upper right first molar teeth with 5–0 silk sutures (OrhanBoz AS, Ankara, Turkey) that were knotted at the vestibule under 10 mg/kg xylazine (Rompun®, Bayer, Topkapı, Turkey) and 40 mg/kg ketamine (Ketalar, Pfizer, Istanbul, Turkey) intramuscular anesthesia (IM). Ligature placement around the posterior teeth induced local bacteria accumulation that led to inflammation and bone loss (28). The ligatures were removed on the 30th day to allow for healing of the inflammation and healing response. After these procedures, the animals were examined for periodontitis (29).

***Study design***

 30 rats were randomly divided into three groups of 10: control group (C), saline was administered by gavage; experimental periodontitis group (P), after the removal of the ligature that was attached to induce periodontitis, saline was administered by gavage; crocin-experimental periodontitis (Cr-P) group, after the removal of the ligature, 100 mg/kg/day Cr (Sigma Chemical Co., St. Louis, MO, USA) was administered. We dissolved 100 mg/kg Cr in saline daily and administered it by gavage to the Cr-P group for 15 days. The C and EP groups were given saline for 15 days. All treatments were 1 ml and were administered at the same time every day. 

***Blood and tissue specimen pooling***

After the laparotomy, kidney tissues and blood samples were collected. The animals were then euthanized with a high-dose anesthetic. The left kidney samples were transferred into 10% formaldehyde for histopathological analysis, and the right kidney tissues were stored at −80 °C until biochemical analysis. 

***Biochemical analysis***

The kidney tissues were transferred from the deep-freeze storage. Frozen kidney tissues were weighed immediately for malondialdehyde (MDA), reduced glutathione (GSH), superoxide dismutase (SOD), catalase (CAT) analysis, total anti-oxidant status (TAS), total oxidant status (TOS), oxidative stress index (OSI), and protein levels. 10% homogenates were obtained with phosphate buffer supplementation and the samples were homogenized at 12,000 rpm for 1–2 min in ice (IKA, Germany). The MDA levels were determined with these homogenates. The supernatant was obtained after the homogenates were centrifuged at + 4 °C and 5000 rpm for 30 min. The supernatants were used to determine GSH, SOD, CAT, TAS, TOS, OSI, and protein levels. 

***Determination of the MDA level***

The method developed by Ohkawa *et al*. (30) was used in MDA analysis. MDA concentration was measured with the absorbance of the homogenates extracted from the n-butanol phase of the pink-colored product of MDA and thiobarbituric acid reaction at 535 and 520 nm at 95 °C. Results were presented as nmol/g wet tissue.

***Determination of the GSH level***

GSH analysis was conducted with the method proposed by Ellman (31). In this method, the glutathione and 5,5′-dithiobis 2-nitrobenzoic acid (DTNB) reaction causes a yellow-green color. The reduced glutathione content was determined based on the intensity of the final color at 410 nm. Results are presented in nmol/g wet tissue.

***SOD activity measurement***

Sun *et al*. (32) methodology was employed to measure the SOD enzyme activity, where superoxide radicals are generated by xanthine-xanthine oxidase. The superoxide radical turns into a blue formazan due to NBT (nitro blue tetrazolium) reduction. SOD activity could be measured by formazan absorbance at 560 nm. Results are presented in U/g protein.

***CAT activity measurement***

The Aebi and Bergmeyer method was employed to measure CAT enzyme activity (33). The method entails the hydrogen peroxide (H_2_O_2_) induced absorbance in the ultraviolet spectrum. The maximum absorbance is observed at 240 nm. When hydrogen peroxidase is added to the medium, catalase breaks it down into water and oxygen, and the absorbance is reduced at 240 nm. The decrease observed in absorbance within 1 min is employed to measure the enzyme activity. Results are presented in K/g protein.

***Protein level measurement***

The total protein content of kidney tissues was determined with the Lowry *et al*. (34) method at 700 nm. Results are presented in mg/ml.

***TOS measurement***

Erel’s (35) method was employed to determine TOS. It was measured based on the kit instructions (Rel Assay Diagnostics, Gaziantep, Turkey). Results are presented in micromole H_2_O_2_eqv.

***TAS measurement***

Erel’s (36) method was employed to determine TAS. It was measured based on the kit instructions (Rel Assay Diagnostics, Gaziantep, Turkey). Results are presented in milimol Trolox eqv.

***Oxidative stress index measurement***

OSI was calculated using the method described by Erel (36). OSI was calculated based on the kit instructions (Rel Assay Diagnostics, Gaziantep, Turkey). OSI is equal to the division of TOS by TAS: OSI (arbitrary unit) = TOS (μmol H_2_O_2_eqv/1) / TAS (mmol Trolox eqv/l) x 10. Results are presented in arbitrary unit (AU).

***Biochemical serum analysis***

Blood samples were centrifuged at 600 x g and 4 °C for 20 min to obtain the serum. The analysis kits (Abbott, Abbott Park, Illinois, USA) and the autoanalyzer enzymatic colorimetric method (Architect C8000) were employed to determine serum urea and creatinine levels. Tumor necrosis factor-α (TNF-α), interleukin-6 (IL-6) and interleukin 1β (IL-1β) levels were measured with a high-sensitivity ELISA kit for rats (Immuno-Biological Laboratories Co., Ltd., Takasaki-Shi, Japan) based on the manufacturer instructions.

***Histological analysis***

The kidney tissues were set in 10% neutral-buffered formaldehyde for 48 hr. They were then dehydrated in ethanol series (50%–99%) and cleared in xylene series. The product was embedded in paraffin wax and 6 µm thick paraffin sections were obtained and placed on slides and stained with hematoxylin-eosin (H-E) and Periodic acid–Schiff (PAS). All sections were examined under a light microscope (Nikon Eclipse Ni-U) with a camera (Nikon DS-Fi3). The microscopic images were analyzed with the image analysis system (Nikon NIS-Elements Documentation 5.02) (Nikon Corporation, Tokyo, Japan).

***Histological examination***

The dimensions of 100 random glomeruli were measured in each section to determine the mean glomeruli dimension. Kidney damage score was calculated with glomerular, interstitial inflammation, and tubular epithelium and tubular basement membrane damage change measurements. Glomerular damage (capillary collapse, narrowing, or removal of the Bowman’s space) was scored as follows: 0- normal appearance; 1- mild collapse; 2- moderate collapse; 3- severe collapse of the affected glomeruli (maximum total glomerular collapse score = 300). The tubular epithelium damage (heterochromatic or pycnotic nucleus, irregularity in cell borders, breakdown and shedding of cells) was scored as follows: 0- absent; 1- <25% of tubules injured; 2- 25–50% of tubules injured; 3- >50% of tubules injured. The inflammation (accumulation of leukocytes) was scored as follows: 0- absent; 1- mild; 2- moderate; and 3- severe. The tubular basement membrane damage (irregularity or discontinuous of the basement membrane) was scored as follows: 0- absent; 1- <25% of tubules injured; 2- 25–50% of tubules injured; 3- >50% of tubules injured in PAS stained sections (maximum total score = 9).

***Statistical analysis***

Normal distribution of the data was determined with the Shapiro-Wilk test. Normally distributed data were summarized by mean and standard deviation. The homogeneity of group variances was determined using the Levene test. If the variances of the groups were found to be homogeneous, one-way analysis of variance and Tukey HSD *post-hoc* test, otherwise Welch test and Tamhane’s T2 *post-hoc* test were used. Histological scores are presented as median, minimum-maximum values and group comparisons were conducted by Kruskal-Wallis and Conover pairwise comparison methods. In all analyses, two-sided significance level was accepted as *P*<0.001.

## Results

***Biochemical results***

It was determined that MDA, TOS, OSI levels were higher and GSH, SOD, CAT, and TAS levels were lower in group P kidney tissues when compared with all other groups. We found that high MDA, TOS, and OSI levels in the P group decreased in the P + Cr group, while low GSH, SOD, CAT, and TAS levels in the P group increased and approached the C group levels (Table 1). We observed that Bun, creatinine, TNF-α, IL-6, and IL-1β levels were higher in group P serum samples when compared with all other groups. We found that the high P group serum levels approached the C group levels in the P + Cr group (Table 2).

***Histological results***

**Figure 1:**
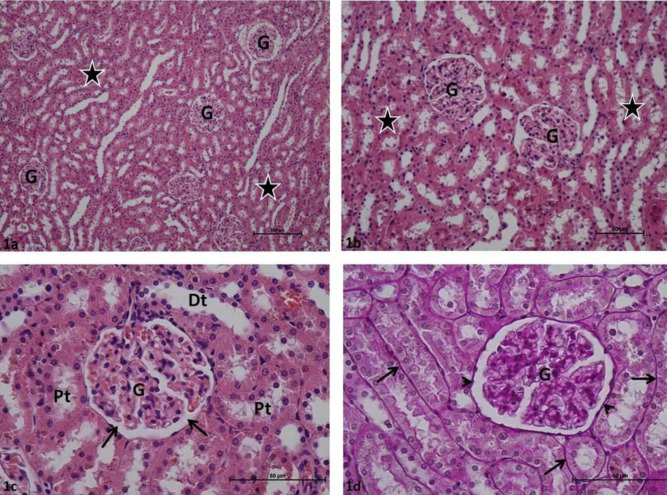
a: Control group: Glomeruli (G), renal tubules (asterisks). H-E, x10; b: Control group: Glomeruli (G), renal tubules (asterisks). H-E, x20; c: Control group: Glomeruli (G), Bowman's space (arrow), distal tubule (Dt), proximal tubule (Pt). H-E, x40; d: Control group: Glomeruli (G), renal tubule basement membrane (arrow), Bowman capsule basement membrane (arrow). H-E, x40

**Figure 2:**
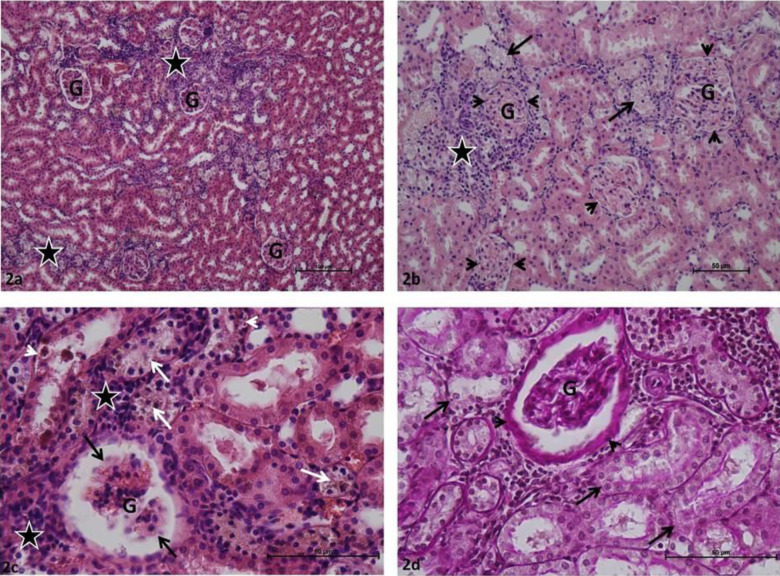
a: Periodontitis group: Glomeruli (G), inflammatory cell infiltration (asterisk). H-E, x10; b: Periodontitis group: Glomeruli (G), Bowman's space obstruction (arrowhead), tubular necrosis (arrow) inflammatory cell infiltration (asterisk). H-E, x20; c: Periodontitis group: Glomeruli (G), Glomerular capillary necrosis (black arrow), inflammatory cell infiltration (asterisk), tubular necrosis (white arrow), tubular epithelial damage (arrowhead). H-E, x40; d: Periodontitis group: Glomeruli (G), renal tubule basement membrane damage (arrow), thickened Bowman's capsule basement membrane (arrowhead). H-E, x40

**Figure 3:**
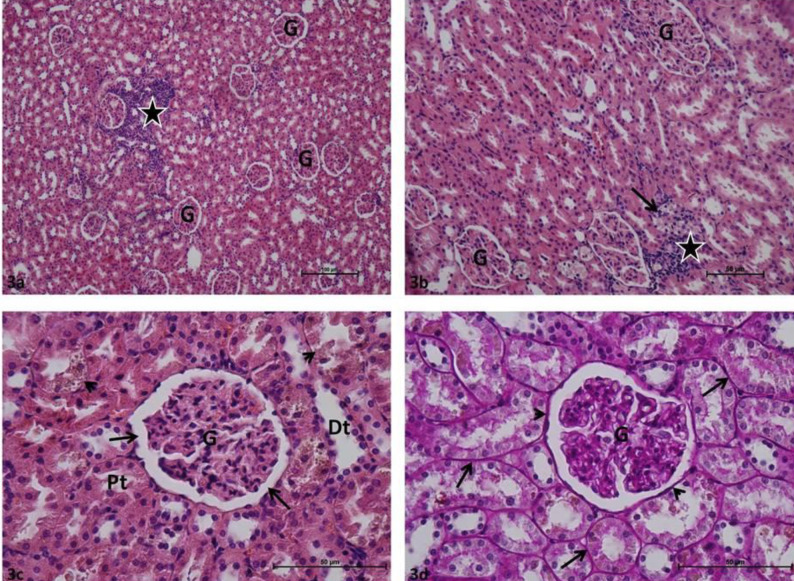
a: Periodontitis + Crocin group: Glomeruli (G), inflammatory cell infiltration (asterisk). H-E, x10; b: Periodontitis + Crocin group: Glomeruli (G), inflammatory cell infiltration (asterisk), tubular epithelial damage (arrow). H-E, x20; c: Periodontitis + Crocin group: Glomeruli (G), Bowman's space (arrow), distal tubule (Dt), proximal tubule (Pt), tubular epithelial damage (arrowhead). H-E, x40; d: Periodontitis + Crocin group: Glomeruli (G), renal tubule basement membrane (arrow), Bowman's capsule basement membrane (arrowhead) H-E, x40

Glomeruli and renal tubules in group C rat kidney sections were determined in normal histological structure. Bowman intervals were clear. In Periodic acid – Schiff-stained samples, the basal membranes of the glomerular and renal tubules were thin, continuous, and PAS + stained (Figure 1). Various degrees of Glomerular collapse and local glomerular capillary necrosis were commonly identified in P group rat kidney sections. It was observed that Bowman’s spaces were completely absent in certain glomeruli. Inflammatory cell infiltration was common in periglomerular and peritubular interstitial tissue in most kidney sections. Various degrees of tubular epithelial damage and localized tubular necrosis were observed in renal tubules. High PAS + density was observed in glomerular capillary basement membranes, marked thickening was observed in Bowman’s capsule basement membranes, obstruction and degeneration were observed in basement membranes of renal tubules in preparations stained with Periodic acid-Schiff (Figure 2). Minimal glomerular collapse, epithelial damage in renal tubules, and inflammatory cell infiltration in the interstitial area were common in P + Cr group rat kidney sections. The damage score was lower when compared with that of the P group. In most Periodic acid-Schiff-stained preparations, the basal membranes of the glomerular and renal tubules were thin and continuous, PAS + stained, and had normal histological appearance (Figure 3).

Glomerular dimensions were reduced and % Glomerular collapse was higher in the P group than in all other groups (Table 3). Histological error score analysis of the P group revealed that interstitial inflammation, tubular epithelium injury, tubular basement membrane injury, and total damage score were higher than in all other groups (Table 4). In the P + Cr group, all these parameters approached the C group levels when compared with the P group.

**Table 1:** Kidney tissue oxidant-antioxidant parameters

	**C** (n=10)	**P** (n=10)	**P+Cr** (n=10)	**P**
**MDA** (nmol/gwt)	871,11±20,51^a^	1223,77±41^b^	987,92±40,85^c^	<0,001
**GSH **(nmol/gwt)	1034,77±27,22^a^	840,1±35,52^b^	959,52±26,55^c^	<0,001
**SOD** (U/g protein)	26,11±2,55^a^	8,77±1,9^b^	15,49±2,29^c^	<0,001
**CAT **(K/g protein)	16,26±1,77^a^	7,76±1,14^b^	11,37±1,77^c^	<0,001
**TAS **(mmol/L)	1,81±0,09^a^	1,12±0,05^b^	1,34±0,08^c^	<0,001
**TOS **(µmol/L)	25,39±2,38^a^	44,48±4,84^b^	32,95±3,23^c^	<0,001
**OSI** (A/U)	144,49±16,92^a^	420,08±51,77^b^	256,23±34,71^c^	<0,001

MDA: malondialdehyde; GSH: reduced glutathione; SOD: superoxide dismutase; CAT: catalase; TAS: total anti-oxidant status; TOS: total oxidant status; OSI: oxidative stress index. Groups: C: control; P: periodontitis; P-Cr: periodontitis + crocin. In each row, significant difference (*P*<0.001) is denoted with different superscripts

**Table 2:** Anti-inflammation and serum biochemical parameters

	**C** (n=10)	**P** (n=10)	**P+Cr** (n=10)	**P**
**Tnf-α **(pg/mL)	30.1±1^a^	50±1.2^b^	34.6±1^c^	<0.001
**IL-6 **(pg/mL)	349.2±1.9^a^	592.9±7.7^b^	396.9±5.7^c^	<0.001
**IL-1β **(pg/mL)	150±5.6^a^	247±6.1^b^	193.7±7.2^c^	<0.001
**Bun **(mg/dL)	9.7±0.9^a^	23.6±1.3^b^	13.7±0.9^c^	<0.001
**Creatinine** (mg/dL)	2±0.1^a^	4.1±0.2^b^	2.6±0.2^c^	<0.001
				

TNF-α: tumor necrosis factor–α; IL-6: interleukin-6; IL-1β: interleukin-1β. Groups: C: control; P: periodontitis; P-Cr: periodontitis + crocin. In each row, significant difference (*P*<0.001) is denoted with different superscripts

**Table 3:** Histological damage score

	**C** (n=9)	**P **(n=9)	**P+Cr** (n=9)	**P**
**Interstitial inflammation**	0 (0-0)^a^	2 (2-3)^b^	1 (0-2)^c^	<0,001
**Tubular epithelium injury**	0 (0-1)^a^	2 (1-3)^b^	1 (0-1)^c^	<0,001
**Tubular basement membrane injury**	0 (0-0)^a^	2 (1-2)^b^	0 (0-1)^a^	<0,001
**Total damage score**	0 (0-1)^a^	7 (6-9)^b^	2 (1-3)^c^	<0,001

Groups: C: control; P: periodontitis; P-Cr: periodontitis + crocin. In each row, significant difference (*P*<0.001) is denoted with different superscripts

**Table 4:** Glomerular diameter and glomerular collapse

	**C **(n=9)	**P** (n=9)	**P+Cr** (n=9)	**P**
**Glomerular dimension (µm)**	80,6(71,3-86,4)^a^	56,5(48,3-65,8)^b^	65,6(60,4-72,1)^c^	<0,001
**% Glomerular collapse** ** 0**	98(93-100)^a^	27(13-41)^b^	56(44-67)^c^	<0,001
**% Glomerular collapse** ** 1**	2(0-7)^a^	31(21-43)^b^	36(28-41)^b^	<0,001
**% Glomerular collapse** ** 2**	0(0-0)^a^	28(22-51)^b^	6(3-13)^c^	<0,001
**% Glomerular collapse** ** 3**	0(0-0)^a^	11(6-18)^b^	2(0-5)^c^	<0,001

Groups: C: control; P: periodontitis; P-Cr: periodontitis + crocin. In each row, significant difference (*P*<0.001) is denoted with different superscripts

## Discussion

It could be suggested that P is a common chronic inflammatory disease in humans and affects about 40–50% of the global population. Severe P is the sixth most common human disease (11.2%) (37). The global impact of P increased by 57.3% between 1990 and 2010 (38). P causes significant increases in both acute-phase reactants (CRP, IL-6) (37-39) and oxidative stress biomarkers in plasma (40). The inflammation is not only localized but could also affect the whole body via complex inflammatory responses. Several studies have focused on periodontal and systemic disease interactions including chronic kidney disease (CKD), which is a major public health concern in the USA and other nations (41, 15). 

A study investigated the effects of the rat P model on multiple organs. It reported that P led to high BUN levels in rat serum (42). Another study investigated the effects of the rat P model on kidney tissues. It observed higher MDA levels, lower GSH and serum creatinine levels, morphological changes in the renal cortex, peritubular and glomerular congestion, and widened Bowman’s spaces in P group kidney tissues when compared with the C group (43). Researchers investigated the effects of the mice P model on kidneys for 10 days and reported that serum creatinine and urea levels increased and glomerular and tubulointerstitial pathologic changes were observed in group P kidney samples when compared with group C (44). A study investigated the changes in rat kidney tissues on the 21st day of the apical periodontitis (AP) model. It reported that anti-oxidant status and catalase activities were different in AP group rat kidney tissues when compared with the C group (45). Researchers investigated the impact of the AP model on Wistar rat kidney tissues. It reported that kidney tissue interleukin 17 (IL-17) levels were higher in the experimental group when compared with the C group and observed impairments in kidney tissue histopathology (46). Other researchers studied the effects of periodontal inflammation on rats. They reported that periodontal inflammation led to excessive lipid peroxide content and affected kidney tissues (8-OHdG levels) (47). The present study’s biochemical findings revealed increased kidney tissue MDA and TOS, and BUN, Creatinine, TNF-α, IL-6, and IL-1β levels in P model serum. We also observed a decrease in GSH, SOD, CAT, and TAS levels in kidney tissues. Histopathological analysis revealed an increase in glomerular collapse and local glomerular capillary necrosis, diffuse inflammatory cell infiltration in periglomerular and peritubular interstitial tissue, decrease in glomerular dimension and increase in percentage of glomerular collapse, interstitial inflammation, tubular epithelium injury, tubular basement membrane injury, and total damage score in rat kidney tissues.

Abou-Hany *et al*. administered 20 mg/kg Cr as a protector of streptozotocin-induced diabetic nephropathy (DN) model rats. They found that serum creatinine, BUN, and IL-6 levels and kidney tissue MDA levels increased, GSH and SOD levels decreased, and kidney tissue histopathology deteriorated in the DN group. In the DN + Cr group, they observed biochemical parameters that were close to group C and improvements in kidney tissue histopathology when compared with the DN group (48). In another study, Abou-Hany *et al*. created an ischemia-reperfusion (I/R) model and administered 20 mg/kg Cr to rats for 7 days as a protective. They reported an increase in serum BUN and creatinine levels, increase in kidney tissue MDA levels, decrease in GSH and SOD levels, and deterioration in renal tissue histopathology in the I/R group. They argued that Cr administration led to improvements in biochemical and histopathological findings (49). In a streptozotocin-induced diabetes model (DM), a researcher administered 50 mg/kg Cr as a protective for 21 days. Increased MDA, TOS, and OSI levels, decreased TAS levels, and deterioration in kidney tissue histopathology were reported in the DM group when compared with all other groups. It was reported that Cr administration led to improvements in both biochemical parameters and histopathological findings when compared with the DM group (50). Erdemli *et al*. administered tartrazine (T) and 50 mg/kg Cr as a protective for 21 days and investigated the effects of these administrations on rat kidney tissues. They reported that T administration increased rat serum BUN and creatinine levels, kidney tissue MDA and TOS levels, decreased kidney tissue TAS, GSH, and CAT levels, and led to deterioration in kidney histopathology. They reported that Cr + T administration exhibited improvements in all biochemical parameters and histopathological findings when compared with the T group (23). Rezaee-Khorasany *et al*. administered 5 g/kg ethanol and 10, 20, and 40 mg/kg Cr to rats as a protective agent and investigated the outcomes in kidney tissues. Ethanol administration led to an increase in rat serum BUN and creatinine levels, an increase in kidney tissue MDA, IL-6, and TNF-α levels, a decrease in GSH levels, and deterioration in histopathology. They reported that Cr administration in different doses exhibited positive effects on the ethanol nephrotoxicity in a dose-dependent manner (51). In our study, Cr administration led to improvements in oxidative stress, inflammation parameters, and histopathological findings when compared with the P group, which were consistent with the findings of other studies.

## Conclusion

The present research was the first with the employed variables and demonstrated that P led to inflammation in the whole body. As the inflammation continued, oxidative stress was induced and oxidant content increased, leading to kidney damage. Against the increasing oxidant content, Cr administration improved the oxidant/anti-oxidant balance with the increase in anti-oxidant capacity. The present study aimed to demonstrate the strong anti-inflammatory and anti-oxidant properties of Cr. Cr could play a protective role in P-induced nephrotoxicity.
